# Responses of Tomato Plants under Saline Stress to Foliar Application of Copper Nanoparticles

**DOI:** 10.3390/plants8060151

**Published:** 2019-06-04

**Authors:** Fabián Pérez-Labrada, Elsy Rubisela López-Vargas, Hortensia Ortega-Ortiz, Gregorio Cadenas-Pliego, Adalberto Benavides-Mendoza, Antonio Juárez-Maldonado

**Affiliations:** 1 Doctorado en Agricultura Protegida, Universidad Autónoma Agraria Antonio Narro, Saltillo 25315, Coahuila, México; fabperlab@outlook.com (F.P.-L.); lopez2690vargas@gmail.com (E.R.L.-V.); 2 Centro de Investigación en Química Aplicada, Saltillo 25294, México; hortensia.ortega@ciqa.edu.mx (H.O.-O.); gregorio.cadenas@ciqa.edu.mx (G.C.-P.); 3 Departamento de Horticultura, Universidad Autónoma Agraria Antonio Narro, Saltillo 25315, México; adalerto.benavides@uaaan.edu.mx; 4 Departamento de Botánica, Universidad Autónoma Agraria Antonio Narro, Saltillo 25315, Coahuila, México

**Keywords:** reactive oxygen species, antioxidants, enzymes, fruit quality, nanotechnology, oxidative stress

## Abstract

The tomato crop has great economic and nutritional importance; however, it can be adversely affected by salt stress. The objective of this research is to quantify the agronomic and biochemical responses of tomato plants developed under salt stress with the foliar application of copper nanoparticles. Four treatments were evaluated: foliar application of copper nanoparticles (250 mg L^−1^) with or without salt stress (50 mM NaCl), salt stress, and an absolute control. Saline stress caused severe damage to the development of tomato plants; however, the damage was mitigated by the foliar application of copper nanoparticles, which increased performance and improved the Na^+^/K^+^ ratio. The content of Cu increased in the tissues of tomato plants under salinity with the application of Cu nanoparticles, which increased the phenols (16%) in the leaves and the content of vitamin C (80%), glutathione (GSH) (81%), and phenols (7.8%) in the fruit compared with the control. Similarly, the enzyme activity of phenylalanine ammonia lyase (PAL), ascorbate peroxidase (APX), glutathione peroxidase (GPX), superoxide dismutase (SOD), and catalase (CAT) increased in leaf tissue by 104%, 140%, 26%, 8%, and 93%, respectively. Foliar spraying of copper nanoparticles on tomatoes under salinity appears to induce stress tolerance to salinity by stimulating the plant’s antioxidant mechanisms.

## 1. Introduction

Plants are subjected to numerous stresses during their development that harm their productivity. Salinity is an abiotic stressor of great significance since it affects about 45 million ha of irrigated soils, reducing the quantitative and qualitative production of crops [1]. The ionic species that can induce salinity are NaCl, Na_2_SO_4_, MgSO_4_, CaSO_4_, MgCl_2_, KCl, and Na_2_CO_3_, with NaCl being the most prevalent salt and having a greater effect due to its dissociation into Na^+^ and Cl^−^ [2]. These salts can cause osmotic and/or ionic alterations at the cellular level, interfering with the nutrition of plants and altering their growth and performance [3]. 

Under salinity conditions, plants have two phases of response: a rapid phase (water deficit) and a slow phase (salt accumulation and toxicity) [1]. In both cases, stomatal conductance, transpiration, and CO_2_ availability decrease, and the photosynthetic process is altered. As a consequence, oxidative stress is induced by the increased generation and presence of reactive oxygen species (ROS) and/or reactive nitrogen species (RNS) [4]. Among the main ROS are the free radicals O_2_^•−^ and OH^•^ and the non-radicals H_2_O_2_ and ^1^O_2_ [5,6]. The main RNS include the radicals nitric oxide (NO^•^) and nitrogen dioxide (NO_2_^•^), as well as non-radicals such as nitrous acid (HNO_2_), dinitrogen tetroxide (N_2_O_4_), peroxynitrite (ONOO^−^), and nitroxyl anion (NO^−^), among others [7,8]. Both types of compounds may be present in the chloroplast, peroxisome, mitochondria, apoplast, cell membrane and wall, and endoplasmic reticulum [5,6,9]. 

Both ROS and RNS act as markers in stress conditions since they trigger a non-enzymatic and enzymatic response that allows the plant to tolerate stress. Some of the non-enzymatic compounds are ascorbic acid (ASH), glutathione (GSH), alkaloids, carotenoids, flavonoids, phenolic compounds, and tocopherol [10]. Among the key enzymatic compounds in ROS elimination are superoxide dismutase (SOD, EC 1.15.1.1), catalase (CAT, EC 1.11.1.6), peroxidase (POD, EC 1.11.1.7), ascorbate peroxidase (APX, EC 1.1.11.1), glutathione reductase (GR, EC1.6.4.2), glutathione peroxidase (GPX, EC 1.11.1.7), monodehydroascorbate reductase (MDHAR, EC 1.6.5.4), and dehydroascorbate reductase (DHAR, EC 1.8. 5.1), among others [11,12,13].

The tomato crop is a vegetable of great economic importance for its high nutritional value in the human diet [14]. However, it can be adversely affected by the salinity of the soil or by salinization caused by the excessive use of fertilizers. As an alternative management strategy, the use of nanotechnology has been studied; specifically, nanoparticles (NPs), which are materials <100 nm in at least one dimension, have been increasingly applied [15]. In recent years, NPs such as carbon nanotubes (CNTs), fullerene, SiO_2_, TiO_2_, CeO_2_, Al_2_O_3_, Ag, ZnO, Fe_3_O_4_/Fe_2_O_3_, and CuO [16] have been the most studied.

Several reports have mentioned that one of the forms of action of NPs is the modulation and stimulation of ROS [17] and likely RNS, as well. The above suggests that the use of NPs can induce an increase in enzymatic activity that allows the plant to tolerate salinity. Karami-Mehrian et al. [18] noted that the application of Ag NPs increased the antioxidant enzymatic activity in tomato plants by modulating the NP-induced oxidative stress by reducing the malondialdehyde (MDA) and Na^+^ content as well as the lipid peroxidation of the cellular structures of the plants under saline stress [19]. Haghighi and Pessarakli [20] reported that applying Si NPs to tomatoes improved the photosynthetic process by inducing tolerance to salinity. Similarly, in *Glycine max* L., SiO_2_ NPs alleviated salt stress by increasing the activity of enzymatic and non-enzymatic compounds [21]. El-Sharkawy et al. [22] reported that the application of K_2_SO_4_ NPs on *Medicago sativa* L. improved the physiological response of the plant to salt stress by reducing the leakage of electrolytes, which in turn increased the level of proline and the activity of antioxidant enzymes such as CAT. Moreover, the foliar application of FeSO_4_ NPs (2 g L^−1^) on cultivars of *Helianthus annuus* L. under salt stress improved CAT activity, polyphenol oxidase (PPO), and peroxidase [15]. Meanwhile, Hernández-Fuentes et al. [23] found that foliar application of Cu NPs (250 mg L^−1^) on tomato plants under salt stress modified the accumulation and degradation of bioactive compounds as well as the antioxidant activity of fruits. However, these findings are limited to the effect of NPs on the bioactive compounds of the fruits. After applying 10 mg of Cu NPs to the substrate of tomato plants developed under salinity conditions, there was a positive effect on the tolerance to this stress [24]. Considering the previous results of the application of NPs, especially the observed beneficial effects of copper nanoparticles on the tomato crop, it is reasonable to expect that the foliar application of Cu NPs will stimulate the antioxidant system of the plants and increase their tolerance to saline stress. Therefore, the present work was developed with the objective of documenting the agronomic and biochemical responses of tomato plants subjected to salt stress with the foliar application of copper nanoparticles.

## 2. Results and Discussion

### 2.1. Tomato Crop Growth

The plant height, stem diameter, fresh aerial weight, dry aerial weight, and yield per plant showed significant differences among treatments (Table 1). Under salinity conditions, the growth and development variables were reduced, and the plant’s yield decreased by ≈50%. The negative effects of salt stress on the growth, development, and productivity of plants can be attributed to osmotic, ionic, or nutritional imbalances [1,3]. On the other hand, in tomato plants under saline conditions, the decrease in production can be attributed to fluctuations in the partition of biomass [25] since the energy required to control stress is greater than that required for the generation of biomass.

The foliar application of Cu NPs induced similar behaviors in plant height, fresh and dry weight of aerial biomass, and yield in Control plants. On the other hand, Cu NPs applied to plants under salinity resulted in all variables adopting trends that were statistically analogous to those induced by the NaCl treatment (Table 1); however, the yield increased by 7%. Similarly, Hernández-Hernández et al. [24] mentioned that applying 10 mg of Cu NPs to tomato growth substrate under salinity induced a positive effect on fruit yield. The responses in the growth and development of the plants found with the application of NPs can differ greatly by the type of NP, concentration, form, and stage of application, as well as the biological material used [26,27]. This seems to indicate that Cu NPs generate a better effect on the control of salinity when applied via the root, as reported in [24].

### 2.2. Mineral Content of Tomato Plants

**Figure 1:**
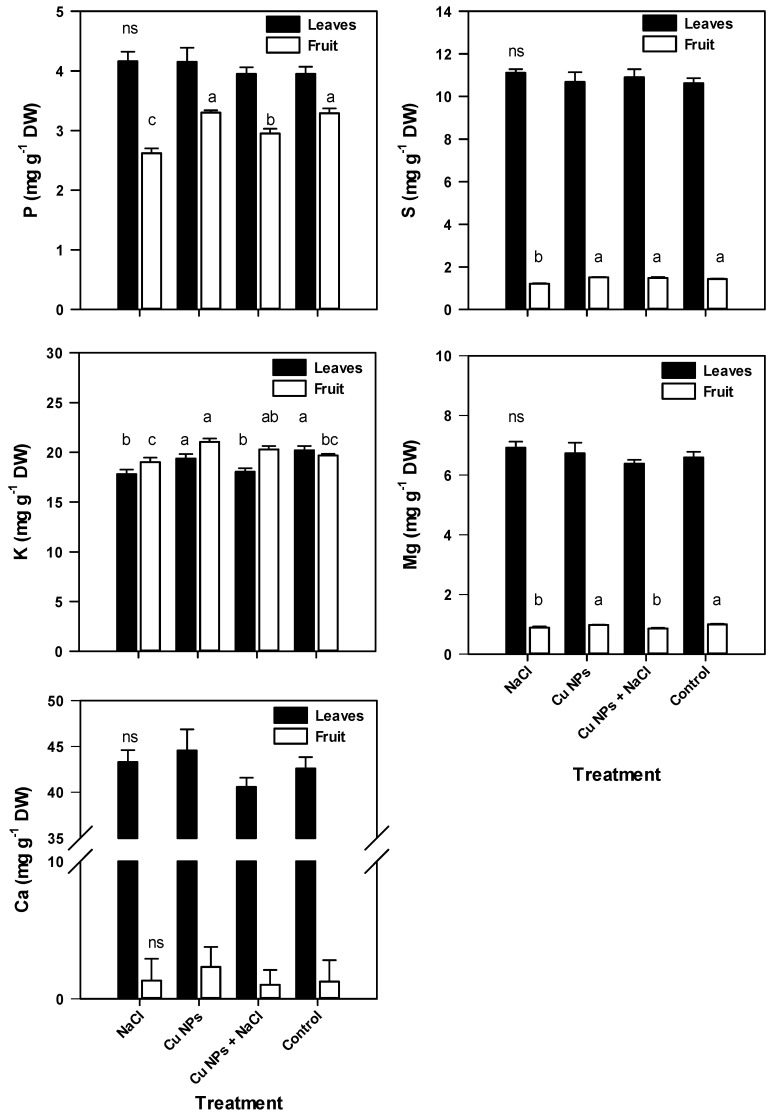
Macronutrient content in leaves and fruits of tomato plants under saline stress and foliar application of Cu NPs. All data are the mean of six replicates ± standard error. Different letters indicate significant differences between treatments according to Fisher’s Least Significant Difference (*p* ≤ 0.05); ns—not significant.

**Figure 2:**
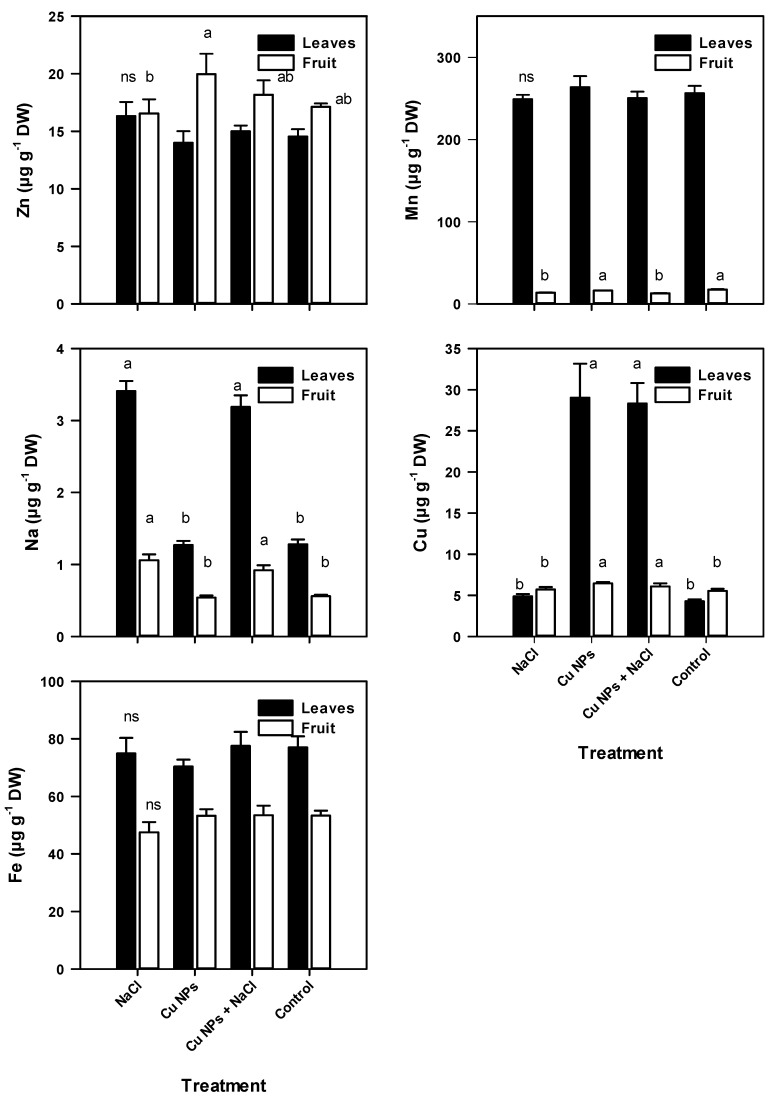
Micronutrient content in leaves and fruits of tomato plants under saline stress and foliar application of Cu NPs. All data are the mean of six replicates ± standard error. Different letters indicate significant differences between treatments according to Fisher’s Least Significant Difference (*p* ≤ 0.05); ns—not significant.

Different organs of the tomato plants had significant differences in mineral content, with the exception of Ca and Fe. Higher content of P, Ca, Mg, S, Na, Fe, Cu, and Mn was found in the leaves, while K and Zn were higher in the fruits (Figure 1 and Figure 2). Tomato plants that were developed under stress due to salinity had higher foliar content of P, Mg, S, Na, and Zn (5.3%, 5.0%, 4.6%, 166%, and 12.3%, respectively) and lower content of K (11.8%), Fe (2.7%), and Mn (2.9%). The high Na^+^ content found in the present work can alter the selectivity of the membrane, thereby modifying the nutritional balance of the plant [28] and inducing an increase in the Na^+^/K^+^ ratio, which is linked to salinity tolerance [29]. The strengthening of this relationship in the treatment of salinity stress reveals that tomato plants experience a disruption to tolerate stress. 

On the other hand, the foliar spray of Cu NPs induced an increase in the content of P (5.1%), Ca (4.6%), Mn (2.8%), and Cu (576.9%) and a reduction in the Na, Fe, and Zn content. In plants receiving the Cu NPs + NaCl treatment, the content of S, Na, Fe, Cu, and Zn increased by 2.6%, 149.2%, 0.7%, 560.4%, and 3.2% compared with the Control. In the same treatment, the content of K, Ca, Mg, and Mn was reduced by 10.7%, 4.7%, 3.2%, and 2.3%, respectively. The increase in Na^+^ found in this study differs from the findings by Hernández-Hernández et al. [24], who reported a reduction in the foliar Na^+^ content of tomatoes developed while exposed to salinity (100 mM) after applying 10 mg of Cu NPs to the growth substrate. This discrepancy may be due to the stage and route of application of NPs as well as the degree of salinity stress. 

The high concentration of Na^+^ observed in the leaf tissue of tomato plants under salinity conditions with the application of Cu NPs may be due to an increase in the compartmentalization of this ion in cellular vacuoles accompanied by an increase in the cytoplasmic K^+^ concentration to achieve osmotic equilibrium [2] and allow the optimal operation of the leaves. Torabian et al. [15] suggested that the accumulation of Fe has a positive relationship with tolerance to salt stress. As a transition metal, Cu can function similarly, making the antioxidant mechanisms more efficient when applied as NPs.

The fruits obtained from plants under conditions of stress by NaCl had lower P, K, Mg, S, Fe, Zn, and Mn contents and higher Ca, Na, and Cu (2.6%, 89.3%, and 3.1%, respectively) compared with the Control. An increase of 0.3%, 7.0%, 5.6%, 16.6%, and 16.5% in the content of P, K, S, Cu, and Zn was found in plants that were treated with foliar spraying of Cu NPs. The plants treated with Cu NPs + NaCl had a lower content of P, Ca, Mg, and Mn and a higher content of K, S, Na, Fe, Cu, and Zn. 

In particular, the content of Cu was higher in the foliar tissue compared with that in the fruit, which suggests a higher accumulation of Cu in the leaves because they were directly exposed to the NPs, whereas there was less translocation of Cu to the fruit. It appears that the ion concentration increased in the tissue exposed to the nanoparticle, as reported by Mosa et al. [30], who developed plants of *Cucumis sativus* hydroponically and found a greater accumulation of Cu in the root tissue compared with the leaves when applying 50, 100, and 200 mg L^−1^ of Cu NPs.

### 2.3. Content of Photosynthetic Pigments

Table 2 shows the chlorophyll content, in which some significant differences were found, in the leaves of tomato plants. Little variation in the content of these pigments between treatments was observed. The foliar application of Cu NPs produced significant differences from the Cu NPs + NaCl treatment, with lower chlorophyll b content observed in plants treated with Cu NPs + NaCl. On the other hand, chlorophyll a and b and total chlorophyll were higher in plants receiving only the NaCl treatment compared with those receiving the Cu NPs + NaCl treatment. The above differs from the findings reported by Hernández-Hernández et al. [24], who cited an increase in the content of photosynthetic pigments after applying Cu NPs (100 mg) to tomato plants subjected to salt stress. The same treatment (Cu NPs + NaCl) reduced the average values of the chlorophylls compared with the Control treatment. Such alterations can be the result of oxidative stress in the photosynthetic apparatus due to the accumulation of Cu from the NPs [30]. Generally all metals inhibit the protochlorophyllid reductase enzyme by preventing the reduction of protochlorophyllide to chlorophyll [31] derived at the high redox potential of metals, including Cu [32]. In this sense, the application of high doses of Cu NPs, in addition to its high reaction surface, could inhibit a greater proportion of the activity of said enzyme by showing a greater redox potential. AlQuraidi et al. [33] report that in plants of *Coriandrum sativum* applying low levels of Cu NPs (200 mg L^−1^) they did not find a significant decrease in the chlorophyll content but with 400 and 800 mg L^−1^ of Cu NPs a significant decrease in the contents of these pigments (chlorophyll a and b).

Plants developed under optimal conditions had a relatively low chlorophyll a/b ratio, whereas plants exposed to NaCl stress had an increased ratio that was likely due to the imbalance in photosynthesis due to excess Na^+^ in the leaves [34]. In that sense, the application of Cu NPs on plants under stress weakened the relationship between chlorophylls and could have improved the photosynthetic rate.

### 2.4. Tomato Fruit Quality

In the fruits of the tomato plants, there were significant differences in firmness, pH, electrical conductivity, total soluble solids, titratable acidity, and oxide reduction potential (ORP). There was an increase in firmness, pH, and electrical conductivity (EC) with the application of Cu NPs compared with these variables in the Control plants. Fruits from plants grown under NaCl had higher electrical conductivity, total soluble solids, and titratable acidity, as well as higher ORP (Table 3). The increase in the electrical conductivity, total soluble solids, and titratable acidity in tomato fruits grown under salinity conditions may be due to the fact that this stress induces the accumulation of soluble sugars and organic acids that counteract the ionic imbalance [35,36]. Campos et al. [37] mentioned that total soluble solids in tomatoes increased in saline conditions as a result of decreased importation of water to the fruit, thus generating an accumulation of solutes in the pulp of the fruit. The application of Cu NPs improved the quality-related variables, and this is likely because a greater proportion of the aforementioned compounds were compartmentalized in the fruit to act as osmoregulators.

Zhao et al. [38] mention that >95% of Cu (provided via foliar as Cu(OH)_2_ NPs ~50 at 1000 nm) is sequestered in the leaves and <5% of Cu is translocated to the lettuce root, this could be an answer of why the foliar exposure of the NPs showed greater accumulation of Cu in the leaf tissue with respect to the fruit. The same authors suggest that NPs can react with the components of the cuticle, the phyllosphere and with the foliar exudates which could form weak acids in the presence of water accelerating the dissolution of Cu(OH)_2_ NPs and therefore capturing and translocating ions Cu towards other tissues. This phenomenon could occur in smaller proportion in the surface of the fruit together with a lower rate of uptake via stomata. In this sense, foliar application is an alternative that can diminish the toxicity of the NPs in the plants derived from the interactions between the cuticle of the leaf-fruit and the NPs. In turn, the translocation of the NPs inside the leaf towards the drains, via phloem, can lead to modifications in its oxidation state [39] without reaching the fruit in large quantity. Finally López-Vargas et al. [40] demonstrated in a previous study that the foliar application of 250 mg L^−1^ of Cu NP did not present a risk of toxicity in tomato fruits.

### 2.5. Antioxidant Compounds of Tomato Plants

**Figure 3:**
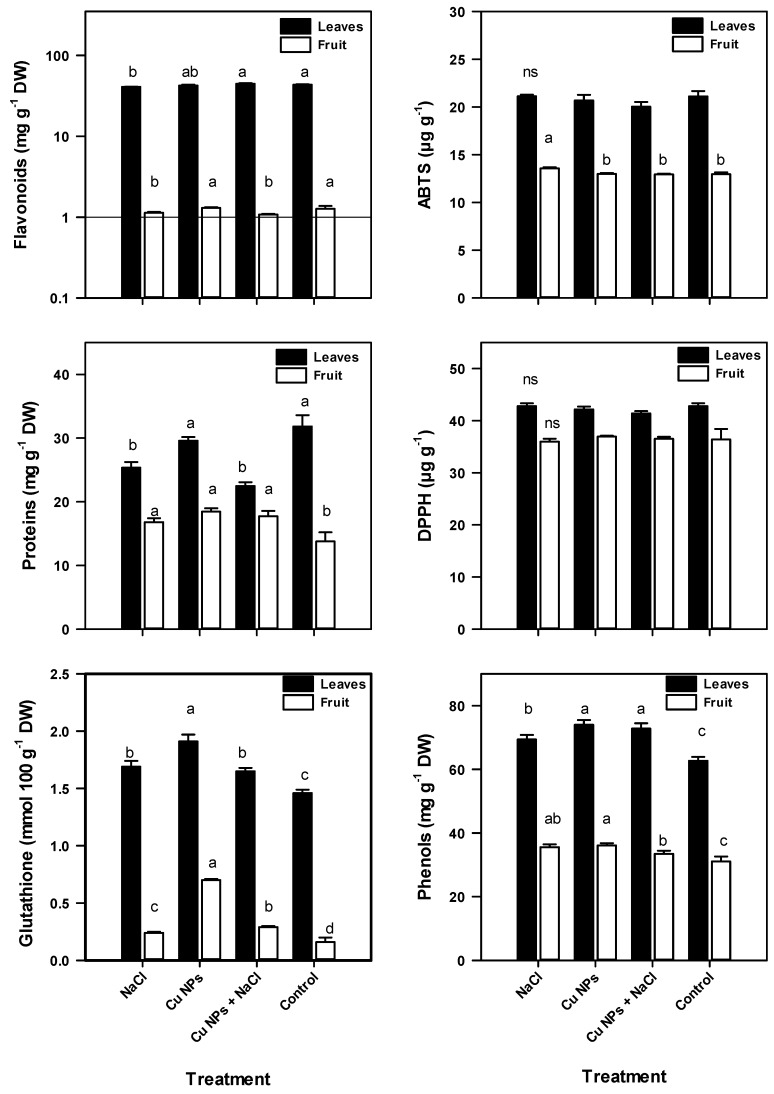
Proteins and antioxidant compounds in the leaves and fruits of tomato plants under saline stress and treated with foliar application of Cu NPs. All data are the mean of six replicates ± standard error. Different letters indicate significant differences between treatments according to Fisher’s Least Significant Difference (*p* ≤ 0.05); ns—not significant.

**Figure 4:**
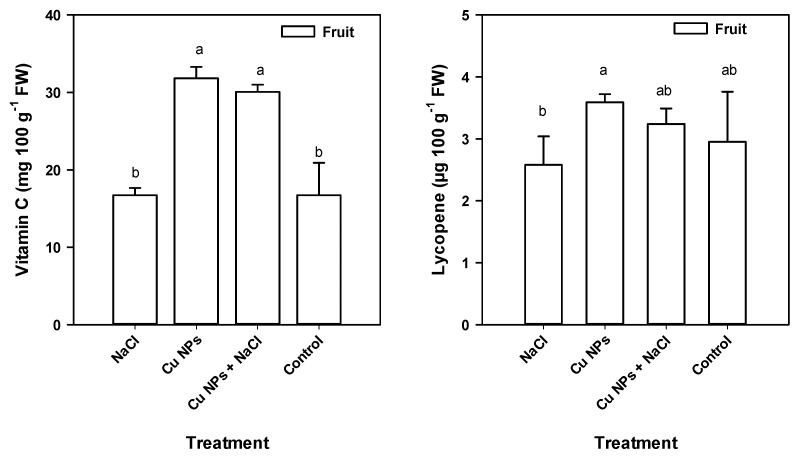
Vitamin C and Lycopene in the fruits of tomato plants exposed saline stress and treated with foliar application of Cu NPs. All data are the mean of six replicates ± standard error. Different letters indicate significant differences between treatments according to Fisher’s Least Significant Difference (*p* ≤ 0.05); ns—not significant.

With the exception of the antioxidant activity by DPPH (1,1-difenil-2-pricrilhidrazil), significant differences in the other quantified antioxidant compounds were found between the leaf tissue and fruits. The foliar tissue had a higher content of GSH, total phenols, and flavonoids, as well as greater antioxidant activity by DPPH and ABTS, compared with the tomato fruit (Figure 3 and Figure 4). 

Salinity stress can induce the generation of ROS and RNS as a signaling response [34,41]. Similarly, Cu NPs can contribute to oxidative stress by increasing H_2_O_2_ and MDA because Cu catalyzes the overproduction of ROS by Haber–Weiss and Fenton reactions [30]. These molecules are triggered in response to the production of enzymatic and non-enzymatic antioxidant compounds [3,41]. The application of Cu NPs induced an increase in the content of vitamin C and lycopene in the fruits compared with their levels in the Control treatment (Figure 4). On the other hand, in salinity-treated plants, the Cu NPs increased the vitamin C and lycopene content by 80% and 25%, respectively. Vitamin C or ascorbic acid is generated by the Smirnoff–Wheeler pathway and is characterized by its high capacity as an electron donor in various enzymatic and non-enzymatic reactions; thus, vitamin C contributes to the detoxification of ROS by reducing them to H_2_O_2_ and then to H_2_O through the APX reaction [5,19,42]. It can also prevent photo-oxidation by means of the pH-mediated modulation of PSII activity and its regulation of zeaxanthin [5]. Since the plants respond in a systemic way to stress and since vitamin C is an antioxidant of great importance, a constituent of the redox state of the cell, its increase in the fruit with the application of Cu NPs (besides being stimulated by the characteristics of the NPs) could be due to an increase in enzymatic activity dehydroascorbate reductase which in turn improved the ratio of the reduced and oxidized forms of ascorbic acid [43]. As the vitamin C-GSH molecule is of great importance in the antioxidant system of the plant, the foliar application of Cu NPs by increasing the concentration of cellular ROS could cause a parallel increase in the assimilation of S and therefore higher presence of GSH, remembering that GSH is the main compound that contains S [44].

Carotenoids such as lycopene, on the other hand, may exert antioxidant activity by preventing the formation or promoting the elimination of ^1^O_2_ or by the xanthophyll cycle [5]. In light of these details, the increase in the content of these compounds by the application of Cu NPs could suggest that the stimulation of these compounds contributes to the mitigation of salt stress in tomato plants.

The content of GSH was increased by 13% in the leaves of tomato plants treated with Cu NPs + NaCl (compared with the Control) and 15% in plants under conditions of salinity. Very similar GSH concentrations were observed between plants treated with Cu NPs + NaCl and those treated with NaCl only, while the GSH content was lower in the Control plants. There was a considerable increase in GSH (337%) in the fruit obtained from plants with application of Cu NPs, and an 81% GSH increase was found in the fruit of plants under salinity conditions with the application of Cu NPs. Only a 50% GSH increase was observed in fruits under stress conditions without Cu NP treatment. The above suggests that the synthesis of GSH could be a mechanism by which plants tolerate salinity stress since this non-enzymatic antioxidant has great versatility due to its high reducing potential, by which it eliminates H_2_O_2_, ^1^O_2_, OH^•^, and O_2_^•−^ [5], in addition to its participation in the regeneration of ascorbic acid. 

Total leaf phenols increased in all treatments compared with the Control, and they increased to a greater extent with the application of Cu NPs (18%). The foliar spraying of these nanoparticles on tomato plants under saline conditions led to a higher content of total phenols (5%) compared with plants stressed with NaCl without Cu NPs. A similar response was observed in the fruits, and the application of Cu NPs resulting in the highest levels of these compounds among the treatments, followed by the levels in fruits obtained from plants stressed by NaCl (total phenols increased by 16% and 14% in the two treatments compared with the Control, respectively). Phenolic compounds exert their antioxidant activity in several ways: by eliminating ROS and RNS by their ability to donate hydrogen from their hydroxyl groups, by inhibiting radical-generating enzymatic activity, or by synergism with other antioxidants [45]. On the other hand, these compounds can also act as pro-oxidants and increase the levels of free radicals by chelating heavy metals and maintaining or increasing their catalytic activity [46]. This detail suggests that the foliar application of Cu NPs could exacerbate said radicals by this mechanism; in both cases, phenolic compounds are important components of the antioxidant system of the plant.

The Cu content increased in plants receiving the Cu NPs + NaCl treatment (2.8%), and it decreased by 2% in plants treated with Cu NPs and 6% in plants exposed to NaCl. A comparison of the behavior of flavonoids in plants under salinity with and without Cu NPs revealed that the application of Cu NPs improved the flavonoid content by 9%. In the fruits of the treatment plants, the application of Cu NPs led to a higher flavonoid content, whereas the flavonoids in plants under salinity conditions (with or without the application of Cu NPs) had an average decrease of 13% compared with the Control treatment. The stimulation of flavonoids (flavonols, flavones, isoflavones, and anthocyanins) by the action of Cu NPs provides a secondary route to stress tolerance at the antioxidant level by eliminating H_2_O_2_ or ^1^O_2_ [5]_._ The preponderance of these compounds in the complementation of other types of antioxidant enzymes [47] plays a vital role in the antioxidant defense system in plants under salt stress. 

Owing to the different mechanisms of enzymatic activity that can occur in vegetal tissue, the ABTS assay, which evaluates antioxidant activity on the basis of the radical cation ABTS and the uptake capacity, was performed [48]. It differs from the DPPH radical technique, which measures antioxidant activity by combining the antioxidants and analyzing the elimination of DPPH radicals [49]. In this regard, it was possible to appreciate that the antioxidant activity in the leaves determined by DPPH and ABTS was similar between the NaCl-treated and Control plants. The lowest activity was found in plants under salt stress with the application of Cu NPs, and the second lowest activity was observed in plants treated with Cu NPs. DPPH and ABTS behaved differently in the fruits: antioxidant activity by DPPH increased in the fruit of plants treated with Cu NPs and decreased in those exposed to salinity (−1.2%) while the activity by ABTS increased (4.7%). Pinedo-Guerrero et al. [50] reported an increase in the antioxidant content of ABTS and DPPH in jalapeno pepper fruits by applying Cu NPs to chitosan–polyvinyl alcohol (Cs-PVA) hydrogels (0.2–10 mg Cu NPs) in the growth substrate. Their observation of increased activity disagrees with the reduction observed in the present work after applying Cu NPs, and the discrepancy is likely caused by interactions between ROS and antioxidants and a decrease in total antioxidant capacity. Although antioxidant enzymatic activities and the ability to capture the DPPH radical are significantly modified in plants subjected to salinity stress [51], the increase in tomato fruit after applying Cu NPs in this work may be due to the capacity of NPs to induce oxidative stress [30].

### 2.6. Antioxidant Activity of Tomato Plants

All enzymatic activity variables showed significant differences. PAL, APX, SOD, and CAT had greater activity in the leaf tissue of tomato plants, while only GPX activity was higher in the fruit (Table 4). The PAL enzyme activity increased in the leaves and fruit in response to all treatments: it increased by 140%, 113%, and 104% in the leaves of plants treated with NaCl, Cu NPs, Cu NPs + NaCl, respectively. In the fruit, the foliar application of Cu NPs on plants with and without salt stress increased the activity of this enzyme by 102% and 108%, respectively. The PAL enzyme catalyzes the deamination of phenylalanine and converts it to cinnamic acid, and this reaction regulates the primary and secondary metabolism [52]. Its increased activity may be due to the damage induced by NaCl. Its increase in leaves may be associated with the biochemical response of the plant to salt stress, and its increase in fruit may be derived from its interaction with NPs.

The foliar application of Cu NPs on plants developed under salinity conditions increased APX (140%), GPX (26%), SOD (8%), and CAT (93%) activity in leaves compared with the Control treatment. In the fruit, there was a decrease in GPX (20%) and SOD (95%) activity in all treatment groups, while APX activity increased after Cu NPs were sprinkled on plants stressed by NaCl. Similarly, catalase activity increased in the fruit of plants in all treatment groups compared with the Control, with the NaCl, Cu NPs, and Cu NPs + NaCl treatments increasing the catalase activity in the fruit by 57%, 157%, and 152%, respectively.

The metalloprotein SOD and its isoenzymes (Mn-SOD, Cu/Zn-SOD, and Fe-SOD) are the first line of defense to reduce the damage caused by ROS, and it is most effective in the dismutation of O_2_^•−^ to H_2_O_2_ and O_2_. This reaction makes the formation of OH^•^ by the Haber–Weiss reaction impossible [5,53]. The resulting H_2_O_2_ is decomposed into H_2_O and O_2_ by APX, CAT, and GPX [13]. When the activity of this enzyme is high, as was the case in the foliar tissue of plants treated with Cu NPs + NaCl, it can increase tolerance to salinity stress.

The APX enzyme removes the H_2_O_2_ that resides in the cytosol and chloroplasts, and it does so efficiently, reducing it to H_2_O and MDHA by using ascorbic acid as a reducing agent [5,9]. The increase in its activity in plants treated with Cu NPs + NaCl could be due to its greater capacity for eliminating the salinity-induced H_2_O_2_ present in the leaf tissue and fruit through the above reduction mechanism, as well as the oxidation of ascorbate by means of the Ascorbate–Glutathione cycle.

On the other hand, the catalase enzyme has high affinity for the H_2_O_2_ molecule and a strong ability to catalyze the dismutation of H_2_O_2_ (mainly in the peroxisome) into water and oxygen without requiring a reducing equivalent; it also reacts with organic peroxides to a lesser extent [5,9,10]. Under salt stress, the plant requires greater generation and expenditure of energy that is provided along with the generation of the H_2_O_2_ radical, which is efficiently eliminated by the catalase enzyme, as evidenced by the increase in its activity in response to NaCl-induced stress and by the probable interaction with the NPs.

GPX reduces H_2_O_2_ or organic hydroperoxides through processes that are mediated by a thiol group and independent of ascorbate. It carries out this reaction by using nucleophiles, such as GSH, thioredoxin (TRX), or glutaredoxins (GRX) [9,10]. Table 4 shows that the application of Cu NPs increased the GPX activity in the foliar tissue of plants under NaCl stress, so this enzymatic activity may be another enzymatic mechanism used by plants to enhance their tolerance to stress by salinity. 

Hernández-Fuentes et al. [23] found that the foliar application of Cu NPs (250 mg L^−1^) on tomato plants under salt stress modified the accumulation and degradation of bioactive compounds, as well as the antioxidant activity of fruits. Similarly, Abdel-Latef [17] reported that applying 0.01% TiO_2_ NPs to *Vicia faba* plants subjected to salinity increased the enzymatic activity of SOD, APX, and CAT. The increase in antioxidant activity in tomato plants under salt stress in response to the application of NPs (Figure 3 and Figure 4, Table 4), especially by an enzymatic mechanism, can improve the retention of K^+^ (Figure 1) by eliminating OH, thus increasing the tolerance to salinity stress [54]. 

The increase in the antioxidant activity induced by the application of Cu NPs found in the present work reaffirms the conclusions by Fu et al. and Huang et al. [55,56], who proposed that NPs stimulate the concentration and activity of antioxidants. This biostimulation may be due to the interaction of NPs with intracellular structures as a result of their high reactivity and ease of penetrating cell barriers [26] by endocytosis, pore formation, transport proteins, or plasmodesmata [27]. The foliar application of NPs induces the generation of ROS and antioxidant compounds more quickly and efficiently since they are absorbed by the porous spaces and pass through the cell wall; once inside, the NPs can bind to the outer side of the cellular membrane by means of electrostatic interactions (with the positive part of the membrane) that favor the reaction with the NPs (negatively charged). Finally, the compartmentalization of the NPs in the cytosol, plastids, vacuole, or nucleus is probably carried out by the potential of the plasma membrane or, to a lesser extent, by endocytosis [57,58,59]. The entrance of NPs induces oxidative stress and, as a consequence, transcriptional reprogramming of the secondary metabolism occurs; that is, enzymatic and non-enzymatic antioxidant mechanisms are activated [58] and increase the plant’s tolerance to salinity stress. It should be mentioned that some of the NPs applied to the leaves of tomato plants can react electrostatically with the metabolites produced by the trichomes on the leaf surface [60].

The increase in enzymatic and non-enzymatic activity mitigates the damage caused by salinity, so these mechanisms are considered stress tolerance pathways in tomato plants and are vital to the cellular defense strategy to counteract the oxidative stress induced by the NPs [18]. Evidently, the enzymatic activity and the non-enzymatic compounds present a complex, interrelated system of antioxidant protection against stress [10,61].

## 3. Materials and Methods 

### 3.1. Crop Growth

The experiment was developed by first establishing the saladette tomato variety “Huno F1” (Harris Moran) of indeterminate growth under greenhouse conditions and soilless culture. The transplant was performed 36 days after sowing in black polyethylene containers with a capacity of 10 L, with a mixture of perlite/peat moss in a 1:1 ratio (*v*/*v*) as the substrate. The culture was managed on a single stem and developed for 100 days after the transplant until fruits were produced for evaluation. A directed irrigation system was used, and Steiner nutrient solution [62] was used as a base to provide the necessary nutrients to the plants. This was applied in all irrigations, and the concentration was modified depending on the phenological stage: a 25% concentration was applied initially, with a 25% increase every two weeks until reaching 100%. The pH was adjusted to 6.5, and the Electrical Conductivity (EC) increased as the concentration of the nutrient solution increased. The EC ultimately reached 2.7 dS m^−1^.

### 3.2. Treatments

The treatments used were a solution with 50 mM of NaCl (T1), Cu NPs at 250 mg L^−1^ (T2), Cu NPs at 250 mg L^−1^ plus NaCl (T3), and a control treatment without the application of Cu NPs or NaCl (T0). The NaCl concentration was selected on the basis of previous works [24,63]. NaCl was added to the solution seven days after the transplant and throughout the development of the crop. Cu NPs applications for T2 and T3 were foliar and carried out 57 and 78 days after transplantation (DAT) using 25 and 35 mL per plant, respectively.

**Figure 5:**
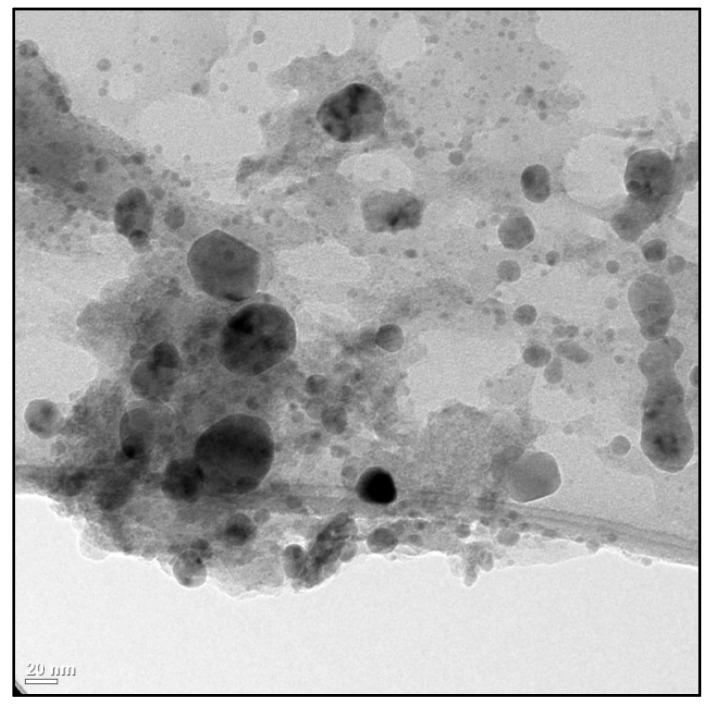
Morphology of copper nanoparticles (Cu NPs) determined by transmission electron microscopy (TEM).

The copper nanoparticles were synthesized in the Research Center for Applied Chemistry, located in Saltillo, Coahuila, México, as described by Juárez-Maldonado et al. [64]. The Cu NPs have a spherical morphology and an average size of 48.3 nm (particle diameters between 20 and 50 nm) (Figure 5). Selection of the Cu NPs size of 50 nm (approx.) was based on previous studies that reported, in addition to a stimulating effect on antioxidant capacity, low or no toxicity [40]. Foliar way was selected because it is an alternative in the uptake of NPs from the leaf surface to the internal tissues by the stomatal route, since the stoma opening is approximately 25 μm long and 3–10 μm wide [65,66,67]. The Cu NPs used in the present work (50 nm) could be captured in greater proportion by the stomatal route. It should be noted that the uptake rate of NPs varies according to leaf morphology, density and stomatal index, hydrophobicity and surface roughness as well as the physiological stage of development of plant organs [67,68].

### 3.3. Agronomical and Biochemical Variables

Agronomic variables of tomato plants were determined (plant height, stem diameter, fresh and dry weight of aerial biomass, and the weight of fruits per plant) to evaluate the effect of treatments on growth and development. For this, 10 plants per treatment were evaluated at the end of the cultivation cycle (100 DAT), with one plant considered one repetition.

The content of mineral elements (P, K, Ca, Mg, S, Na, Fe, Cu, Zn, and Mn) in leaves and tomato fruits was determined with a plasma emission spectrophotometer (ICP, model Thermo Jarrel Ash Irish Advantage 14034000) following the method described in Hernández-Hernández et al. [24]. Two fully expanded leaves (third and fourth) per plant were washed prior to element analysis. The chlorophyll content was determined according to Nagata and Yamashita [69]. Six plants were analyzed per treatment, and the samples were collected at the same time that the fruit samples were taken.

To determine fruit quality, six fruits (one per plant) of uniform size were collected from the third cluster. The tomato fruits had a state of maturity of 6 (light red). The hydrogen potential (pH), electrical conductivity (EC), total soluble solids, firmness, oxide reduction potential (ORP), and titratable acidity (AT) of the fruits were determined following the methods described by López-Vargas et al. [40]. 

Total protein, vitamin C, glutathione (GSH), total phenols, flavonoids, ABTS antioxidant capacity, and the enzymatic activity of ascorbate peroxidase (APX), glutathione peroxidase (GPX), superoxide dismutase (SOD), and catalase (CAT) were determined according to the methods described by López-Vargas et al. [40]. The lycopene content was determined following the method of Nagata and Yamashita [69]. Phenylalanine ammonia lyase (PAL) was determined according to Sykłowska-Baranek [70]. The DPPH antioxidant capacity (1,1-difenil-2-pricrilhidrazil) was determined according to Brand-Williams et al. [71]. 

### 3.4. Statistical Analysis

For the variables evaluated, 10 (agronomical) and six (biochemical) repetitions per treatment were considered, where one plant was considered one repetition. A completely random design was used. Analysis of variance and Fisher’s Least Significant Difference means tests (*p* ≤ 0.05) were performed using Infostat software version 2018 (https://www.infostat.com.ar/).

## 4. Conclusions

From the results of this study, we conclude that tomato plants subjected to salt stress have reduced growth and development as a result of alterations in the Na^+^/K^+^ ratio. The foliar application of copper nanoparticles reduces these alterations by improving the Na^+^/K^+^ ratio.

The foliar application of copper nanoparticles is conducive to better fruit quality. Under salinity conditions, sprinkling the nanoparticles on tomato plants increases the accumulation of antioxidant compounds, such as vitamin C, lycopene, GSH, total phenols, and flavonoids (both in leaves and fruits).

The enzymatic activity of tomato plants under salinity responds positively to the application of copper nanoparticles; specifically, the activity of PAL, APX, GPX, SOD, and CAT increases.

The findings of these experiments suggest that foliar spraying of copper nanoparticles, which stimulates the plant’s antioxidant mechanisms, is a feasible strategy for managing and/or mitigating damage caused by salt stress in tomatoes.

## Figures and Tables

**Table 1:** Agronomic variables of tomato plants under salinity stress with and without the foliar application of Cu nanoparticles (NPs).

Treatment	Plant Height (cm)	Stem Diameter (mm)	Fresh Weight of Aerial Biomass (kg)	Dry Weight of Aerial Biomass (g)	Fruit Weight per Plant (kg)
NaCl	245.78 b ± 1.66	12.07 ab ± 0.15	1.248 b ± 0.045	171.38 b ± 6.73	3.604 b ± 0.197
Cu NPs	263.33 a ± 3.04	12.53 a ± 0.42	1.500 a ± 0.0 52	213.29 a ± 8.58	5.950 a ± 0.121
Cu NPs + NaCl	242.94 b ± 9.25	12.49 a ± 0.19	1.226 b ± 0.040	176.73 b ± 5.38	3.857 b ± 0.132
Control	262.39 a ± 2.82	11.59 b ± 0.15	1.425 a ± 0.045	208.94 a ± 6.73	6.158 a ± 0.197

Different letters in the columns indicate significant differences between treatments according to Fisher’s Least Significant Difference (*p* ≤ 0.05). All data are reported as the mean of 10 replicates ± standard error.

**Table 2:** Chlorophyll content in leaves of tomato plants under saline stress and foliar application of Cu NPs.

Treatment	Chlorophyll a (mg 100 g^−1^ DW)	Chlorophyll b (mg 100 g^−1^ DW)	Total Chlorophyll (mg 100 g^−1^ DW)
NaCl	46.90 a ± 1.94	24.13 a ± 1.41	71.03 a ± 2.97
Cu NPs	34.19 b ± 1.93	22.71 a ± 1.88	56.90 b ± 1.28
Cu NPs + NaCl	25.48 c ± 1.06	13.83 b ± 0.97	39.31 d ± 1.19
Control	46.90 a ± 1.94	24.13 a ± 1.41	71.03 a ± 2.97

Different letters in the columns indicate significant differences between treatments according to Fisher’s Least Significant Difference (*p* ≤ 0.05). All data are the mean of six replicates ± standard error.

**Table 3:** Fruit quality of tomato plants under saline stress and foliar application of Cu NPs.

Treatment	Firmness (kg cm^−2^)	pH	EC (mS cm^−1^)	TSS (°Brix)	TA (% ac)	ORP (mV)
NaCl	3.29 a ± 0.06	4.38 b ± 0.03	4.18 a ± 0.11	6.05 a ± 0.11	0.99 a ± 0.04	147.83 a ± 2.02
Cu NPs	3.46 a ± 0.11	4.48 a ± 0.02	3.66 ab ± 0.23	4.93 b ± 0.05	0.80 b ± 0.04	140.17 b ± 0.83
Cu NPs + NaCl	3.45 a ± 0.08	4.49 a ± 0.02	3.69 ab ± 0.23	6.07 a ± 0.14	0.87 ab ± 0.05	140.67 b ± 1.15
Control	2.85 b ± 0.15	4.37 b ± 0.02	3.33 b ± 0.23	5.15 b ± 0.14	0.94 a ± 0.05	140.00 b ± 1.15

Different letters in the columns indicate significant differences between treatments according to Fisher’s Least Significant Difference (*p* ≤ 0.05). EC—electrical conductivity; TSS—total soluble solids; TA—titratable acidity; ac—ascorbic acid; ORP—oxide reduction potential. All data are the mean of six replicates ± standard error.

**Table 4:** Enzymatic activity in the leaves and fruit of tomato plants with foliar application of Cu NPs and saline stress.

Treatment	PAL (U g^−1^ TP)	APX (U g^−1^ TP)	GPX (U g^−1^ TP)	SOD (U mL^−1^)	CAT (U g^−1^ TP)
**Leaves**					
NaCl	4.72 a ± 0.14	186.29 b ± 1.44	115.32 ab ± 5.23	32.00 bc ± 0.51	675.14 b ± 21.8
Cu NPs	4.17 b ± 0.21	309.30 a ± 7.40	102.60 bc ± 3.63	38.09 a ± 0.51	669.75 b ± 28.1
Cu NPs + NaCl	4.00 b ± 0.08	321.69a ± 6.11	123.99 a ± 4.21	33.56 b ± 0.81	972.89 a ± 50.2
Control	1.96 c ± 0.08	134.14 c ± 4.94	98.39 c ± 4.15	30.95 c ± 1.38	505.13 c ± 39.8
**Fruit**					
NaCl	0.89 b ± 0.06	117.60 c ± 2.25	163.10 b ± 6.09	0.63 c ± 0.06	421.92 b ± 26.5
Cu NPs	1.81 a ± 0.07	137.97 b ± 3.83	157.33 b ± 6.07	4.62 b ± 0.56	691.18 a ± 20.9
Cu NPs + NaCl	1.76 a ± 0.09	181.68 a ± 11.42	194.92 a ± 12.74	3.55 bc ± 0.23	678.10 a ± 37.9
Control	0.87 b ± 0.05	140.03 b ± 4.47	215.48 a ± 8.98	65.65 a ± 2.13	268.80 c ± 21.1

Different letters in the columns indicate significant differences between treatments according to Fisher’s Least Significant Difference (*p* ≤ 0.05). PAL—phenylalanine ammonia lyase; APX—ascorbate peroxidase; GPX—glutathione peroxidase; SOD—superoxide dismutase; CAT—catalase. All data are the mean of six replicates ± standard error.
